# The Impact of an Individual Educational Program on the Quality of Life and Severity of Symptoms of Patients with Irritable Bowel Syndrome

**DOI:** 10.3390/ijerph17124230

**Published:** 2020-06-13

**Authors:** Regina Sierżantowicz, Jolanta Lewko, Grażyna Jurkowska

**Affiliations:** 1 Department of Surgical Nursing, Medical University of Bialystok, 15-089 Bialystok, Poland; renatasierz@wp.pl; 2 Department of Primary Health Care, Medical University of Bialystok, 15-089 Bialystok, Poland; 3 Department of Gastroenterology and Internal Medicine, Medical University of Bialystok, 15-089 Bialystok, Poland; jurkowg@umb.edu.pl

**Keywords:** irritable bowel syndrome, quality of life, education

## Abstract

Background: Irritable bowel syndrome (IBS) is one of the most common functional diseases of the gastrointestinal tract. A typical symptom is changed bowel patterns: diarrhea, constipation, or alternation of the two. Abdominal pains vary in intensity and location, with periods of exacerbation and remission, causing disorganization in everyday life and work. Educational intervention could be one strategy to improve the well-being of IBS patients. Only a few trials have examined this hypothesis. The aim of this study was to examine the effect of an educational program combined with elements of behavioral therapy, individualized for each patient, on quality of life (QOL) and severity of pain of patients with IBS. Methods: In total, 150 IBS patients and 100 healthy persons in the control group were included. QOL (36-Item Short Form Health Survey, SF-36) and pain severity (Visual Analogue Scale) were measured at baseline and six months after education of IBS patients. Results: At baseline, patients with IBS showed highly significantly worse QOL. In the IBS group, significantly higher physical component summary (PCS) and mental component summary (MCS) scores were noted for 35- to 50-year-old patients compared to other patients. Six months after education and behavioral therapy, significant improvement in QOL and a significant decrease in the subjective perception of pain severity were noted compared to values before therapeutic education. Conclusion: An educational program combined with elements of behavioral therapy, individualized for patients with IBS, is an important part of therapy for these patients.

## 1. Introduction

Irritable bowel syndrome (IBS) is one of the most common functional alimentary tract disorders [1]. According to statistics in Western countries [2,3,4,5], IBS symptoms occur in 10–20% of the population, and IBS is twice as frequent in women as in men. This is different in India, where the majority of IBS patients are men [6].

IBS is a combination of chronic abdominal pain associated with a change in the frequency or form of stools. According to Rome Criteria IV, IBS is defined as recurrent abdominal pain on average at least one day per week for the last three months, associated with two or more of the following criteria: related to defecation, associated with a change in the frequency of stools, associated with a change in the form (appearance) of stools [7].

The symptoms usually appear in adolescence and tend to worsen gradually over the years. Symptom onset after the age of 50 is very atypical [8]. The typical symptom is a change in the rhythm of bowel movements: bouts of diarrhea or constipation, or alternating bouts of both. Most patients complain of flatulence and abdominal discomfort, which can be relieved by passing wind or stools [9,10,11]. Patients frequently have upper alimentary tract disorders (gastroesophageal reflux, dyspepsia) coexisting with other diseases (urination disturbances, sexual dysfunction, headaches, fibromyalgia, chronic fatigue) [12].

Patients with typical IBS, with no alarming symptoms, do not require many accessory investigations [12,13]. IBS is characterized by periods of exacerbation and remission, which lead to disorganization of patients’ professional activity. In patients with IBS, infective gastroenteritis could cause systemic inflammation and altered microbiome diversity, which in turn perpetuates a cycle of chronic, low-grade, subclinical inflammation [14]. It is believed that IBS has a negative impact on work efficiency [15,16]. The conducted research confirms that women with IBS more frequently undergo surgical procedures in the abdominopelvic cavity (appendectomy, cholecystectomy, hysterectomy) [16,17,18]. Current research supports a biopsychosocial view of IBS and a holistic approach to the management of IBS symptoms [19].

Although IBS is a mild disorder, the quality of life (QOL) of patients is significantly lower compared to the general population. Studies performed by Mayer et al. [20] and Gralnek et al. [21] showed that the QOL of IBS patients is markedly lower than in the general population and comparable to that of depressive patients. Other authors have observed that the health-related QOL (HRQOL) of IBS patients is similar to or even worse than that observed among patients with organic diseases such as esophageal reflux, asthma, and end-stage renal failure [22,23].

Proper cooperation between doctor and patient, the patient’s awareness of the essence of the disease, and recommendations for lifestyle modification, dietary changes, and physical activity (i.e., widely understood education) [24,25,26] are generally accepted and applied in medical practice. Very few studies have investigated the effect of education on the QOL of patients with IBS. Approximately 50–60% of patients emphasize a relationship between the severity of IBS symptoms and stress, which can be alleviated with nonpharmacological treatment, including psychotherapy, hypnotherapy, relaxation, or lifestyle modification [27,28,29]. A positive effect of some nonpharmacological methods has been confirmed [23]. A multicenter, randomized, open evaluation study published in 2018 found that a health education program improved quality of life and abdominal pain of middle school students with IBS [30].

The impact of a varied educational program on the therapeutic process for patients with IBS has not been evident. It has not yet been explained whether individual education combined with nonpharmacological therapy could contribute to alleviation/remission of symptoms and improved QOL of patients with irritable bowel syndrome.

The study objective was to assess the impact of an educational program with patient-tailored behavioral therapy components on the QOL and severity of symptoms of patients with IBS.

## 2. Material and Methods

The study involved 150 IBS patients (79% women) 18–80 years of age (mean age 48.5 ± 15.77 years) with diagnosed IBS according to the Rome Criteria III treated in the outpatient gastroenterology department [31]. Based on the symptoms, patients were assigned to the IBS group with predominant constipation (60 patients), predominant diarrhea (20), or mixed (70). They were randomly enrolled in the study. All IBS patients remained under the control of a gastroenterologist. Among the co-morbidities, the most frequently reported were allergies, gastroesophageal reflux, cholecystolithiasis, hypertension, and type 2 diabetes.

The control group consisted of 100 subjects who were either healthy or had mild chronic disease, such as mild back pain or mild hypertension, with no complaints suggestive of IBS (80% women, mean age 50 ± 12.03 years). The group was recruited among students, medical workers, and volunteers. Patients in the control group were under the care of a family doctor, and a few were treated for back pain or hypertension.

The educational program, comprising a wide range of issues typical of IBS patients, was tailored for each patient after their knowledge about the disease was assessed (risk factors, diagnostics, therapeutic methods, importance of physical activity, diet modification, the role of stress, methods of stress reduction).

A single therapeutic educational session was conducted by one educational nurse for at least 3 h. All the necessary issues were explained during the session. Moreover, each patient received a packet of written information, including materials routinely used in the outpatient gastroenterology department, prepared based on the literature for the purpose of the educational program. All materials were taken home to be used in the future.

The educational program was conducted individually, after a visit to the gastroenterologist, in a separate room after a prior appointment.

The meeting schedule included the following:

1. A brief conversation about the general well-being of the patient and an assessment of the patient’s knowledge.
2. Making the patient aware of the pathogenesis, symptoms, and treatment of the disease.
3. An indication of recommended lifestyle modifications depending on the dominant form of IBS.
4. A presentation of dietary recommendations depending on the form of the disease (e.g., development of individual meal plans).
5. A presentation of stress management methods (e.g., deep breathing, muscle relaxation).
6. Answers to patient questions.

For one hour after the educational session, the patient was instructed in nonpharmacological methods of IBS therapy: lifestyle modification, dietary changes (e.g., designing a menu), physical activity, and relaxation methods (deep breathing, muscle relaxation, etc.). The patient was encouraged to continue the nonpharmacological therapy in everyday life (physical activity, methods of relaxation, etc.). At the same time, it was emphasized that these nonpharmacological methods should not replace any pharmacotherapy prescribed by a doctor in charge. The estimated cost of the instruction was low. The recommendations concerned dietary modifications according to individual energy assessment of the diet, its composition, and the patient’s eating habits. Nutritional modification was recommended depending on the form and period of the disease. It consisted of eliminating some food products aggravating the ailment, particularly greasy fried foods. Patients were encouraged to limit their consumption of foods that can cause bloating, e.g., legumes, cabbage, onions, and garlic. The need to limit the consumption of natural coffee, strong tea, and alcohol together with avoiding products containing sorbitol and fructose (carbonated drinks, sweets, chewing gum) was pointed out. Patients were motivated to modify their cooking methods during periods of symptom severity; they were recommended to boil, steam, bake in foil, and braise.

### 2.1. Methods of Measurement

A standardized 36-Item Short Form Health Survey (SF-36) was used for subjective assessment of the health-related QOL (HRQOL) of IBS patients and the control group. The survey consists of 36 single questions in eight basic categories. Each category (domain) includes 2–10 questions. Each question may represent one category. The structure of the SF-36 allows for calculating the scores of respective domains, the physical component summary (PCS) and mental component summary (MCS) scores, and the total QOL score. The PCS includes the following domains: physical functioning (PF), role physical (RP), bodily pain (BP), and general health (GH). The MCS contains vitality/vigor/tiredness (VT), social functioning (SF), role emotional (RE), and mental health (MH). The score in each domain ranges from 0 to 100; a higher score means a better QOL. The results were analyzed according to the guidelines designed by the authors of the questionnaire [32].

The intensity of symptoms in IBS patients was determined using a Visual Analogue Scale (VAS) before and after the educational session. The patient marked the severity of symptoms on a numeric scale of 0–10, where 0 indicates no symptoms and 10 the most severe symptoms. Along that scale, slight, moderate, and severe complaints could be marked [33].

All study participants obtained comprehensive oral instructions on completing the questionnaire.

### 2.2. Procedure and Ethical Considerations

The research conformed with good clinical practice guidelines, and the procedures were in accordance with the Helsinki Declaration. All patients signed a consent form to participate in the study. The study was performed from January 2014 to December 2014. The research was approved by the bioethics committee of the Medical University of Bialystok (Resolution no. R-I-002/521/2014).

### 2.3. Statistical Analysis

Statistical analysis was conducted using the Statistica 8.0 package (StatSoft Polska Sp. z o.o., Kraków, Poland) with a Mann-Whitney U-test, Kruskal-Wallis H test, sequence paired Wilcoxon test, chi-square test, and McNemar’s test. Statistical significance was accepted at *p* ≤ 0.05.

## 3. Results

No statistically significant differences were found between the study groups with respect to demographic features. The mean age of patients with IBS was 48.5 ± 15.8 years and that of the control group was 50 ± 12.0 years. Women predominated in both groups. IBS patients were characterized by a slightly higher level of education compared to controls. Among IBS patients, the diagnosis was made within <5 years, less frequently in 5–10 years (33%). Results are presented in Table 1.

Prior to the educational program, the HRQOL of IBS patients and control subjects was assessed using the SF-36. The effect of age and sex of the study subjects on the assessment of QOL in the respective domains was determined.

In the control group, no significant correlation was found for sex and age with the score obtained in the respective domains. In the IBS group, before the educational program, significant differences were observed in the PF, RP, SF, RE, and MH domains between the age groups. After education, highly significant differences were observed in PF, RP, SF, VT, RE, and MH.

Subjectively perceived QOL was also compared in the domains of SF-36 for IBS patients before and after education. A comparison of IBS patients showed highly significant differences in all domains (*p* < 0.01) before and after education. QOL was found to be significantly lower in all SF-36 domains before education, except for one (SF).

IBS patients presented significantly lower QOL scores in the physical sphere compared to the control group. The educational program significantly improved the assessment. Despite falling or rising tendencies observed after education in the respective IBS subgroups, virtually no significant differences were noted between the subgroups with respect to sex, place of residence, form of disease, and its duration before and after education.

Following education, the assessment of QOL by patients aged > 50 years was highly significantly lower in the physical component compared to the other age groups of IBS patients. The results are presented in Tables S1 and S2.

Before education, IBS patients were characterized by highly significantly (*p* < 0.01) lower scores on health in the mental sphere of SF-36. The assessment of QOL was found to be markedly lower in the mental sphere for IBS patients compared to healthy subjects.

A highly significant difference (*p* < 0.01) was found in the distribution of QOL in the mental component before and after education. Generally, following education, patients had a higher assessment of QOL in the mental component. Patients aged < 35 years showed highly significant improvement in QOL scores in the mental sphere after education. Patients aged 35–50 years and >50 years presented lower scores. No differences were found between age groups before education, whereas after education the differences were found to be highly significant (*p* < 0.01). After education, patients aged < 35 years presented highly significantly higher (*p* < 0.01) health assessment in the mental sphere than patients >50 years old. Patients aged 35–50 years showed an intermediate distribution of QOL scores in the mental sphere.

After education, highly significant (*p* < 0.01) improvement was noted in all IBS subgroups. The results are presented in Table 2.

After education, all study patients reported a subjective decrease in the intensity of symptoms (*p* < 0.01). No differences were found for sex, place of residence, form of IBS, and IBS duration before and after education.

No significant correlations were noted between VAS and sex, IBS form, and disease duration. The correlation was highly significant for age before education but not after. Only one patient reported an exacerbation of symptoms from mild to moderate after education. In the remaining cases, no change or evident improvement was observed.

This suggests a positive impact of the educational program, reducing the severity of symptoms. Frequently, the improvement was so evident that the patients changed their pain score, even from maximum severity to no symptoms. Data are presented in Table 3.

## 4. Discussion

Proper assessment of quality of life and the role of nonpharmacological methods in IBS therapy requires the use of suitable research techniques. In our study, the SF-36 was used for this purpose. The severity of intestinal and parenteral complaints in IBS patients was determined using a VAS. The educational–therapeutic program was prepared based on literature guidelines, personal experience of the authors, and cognitive behavioral therapy principles. The research tools so developed allowed a cross-sectional assessment of nonpharmacological methods as well as their impact on the general HRQOL of IBS patients.

IBS is a chronic disease of the alimentary tract that depends on sociocultural factors and other coexisting diseases. It is a cause of 10–15% of visits to family doctors and approximately 25–50% of visits to gastroenterology centers [34]. Outpatient visits are predominant, but hospitalizations and admissions are also common. Due to the chronic nature of IBS, patients frequently return to the doctor in charge, convinced of the need for further investigation to explain the cause of persistent symptoms and expecting effective therapy. Along with recurrent symptoms, work or school performance declines as patients are frequently absent. Their willingness to recover and cooperate with the doctor decreases [35,36].

It has been found that despite accurate diagnosis and proper therapy, the improvement experienced by IBS patients is unsatisfactory, and a reduction in QOL is unexpectedly low compared to the ongoing changes. Four out of five IBS patients complain of having all spheres of life disturbed (physical functioning, social functioning, physical role, bodily pain, emotional role, mental health, vitality, and general health). Although IBS is a mild disorder, health-related quality of life (HRQOL) is significantly lower in these patients compared to patients with some chronic organic diseases [21,23,36,37,38].

In our study, HRQOL in IBS patients was significantly lower compared to healthy control subjects.

We do not know any study results representative of the Polish population in the SF-36 domains that could relate to our IBS study group. Until now, research has revealed statistically significant differences between countries, even those with similar socioeconomic and cultural profiles [22,39,40,41].

In the current study, in all SF-36 domains for the control group (healthy subjects, *n* = 100), the mean values differed from those reported for the population of France standardized for the Encoli study [22], or for the population of Sweden [34], England, or the USA [39]. The results of these studies differed between the respective populations. Thus, it is likely that the population of Poland is also characterized by differences in mean values (median) for the HRQOL domains assessed in the SF-36 questionnaire. The study group of IBS patients was compared with randomly chosen control patients who were considered healthy by a doctor in charge.

The IBS patients had significantly lower scores in all domains but one compared to the control group, irrespective of age and sex. Only in the physical functioning (PF) domain were no differences found between the control group and young patients (<35 years old) with IBS. Lower physical component summary (PCS) and mental component summary (MCS) scores for the overall QOL were noted for the group of IBS patients. The findings showing highly significantly lower QOLs for IBS patients are consistent with those reported by other authors in Europe and the USA [22,23,39,41]. A study from Korea [41] revealed similar differences in Asian countries.

In the Korean study [41], the authors observed highly significantly lower QOL scores in all SF-36 domains for IBS patients compared to the general population, which corresponds with the data obtained in the current study. At the same time, the researchers showed significantly lower QOL scores for women with IBS than men. The results did not indicate any statistically significant sex-dependent differences in QOL of IBS patients. The absolute values were higher in the majority of SF-36 domains for men compared to women, but the difference was not statistically significant. A sex-dependent difference in distribution was noted only in the vitality (VT) domain (*p* = 0.013). No differences were found in the physical and mental components of SF-36 between the groups of women and men with IBS. Education of patients had no impact on the results either.

The difference in QOL depending on the sex of patients observed by the Korean and Polish authors could be ascribed to cultural and social differences between the two countries. Research conducted in Sweden showed significantly lower QOL scores for patients with functional and organic alimentary tract disorders compared to the general population. The differences were sex- and age-dependent. QOL was found to be lower among women with IBS compared to men. Older age was a disadvantage in three out of eight domains of SF-36 (PF, BP, GH). As in our study, the disease duration had no effect on the differences observed. Our findings also indicate a significantly deleterious impact of older age (>50 years) on quality of life compared to younger patients, and as in other studies, no correlation was found with the disease form.

Many researchers have emphasized a significant effect of the severity of symptoms on the subjective assessment of QOL. The findings obtained by Park et al. [41], Creed et al. [42], and Simren et al. [34] showed this relationship.

Our study involved the subjective assessment of symptom severity. No significant correlations were noted between the severity of symptoms and patients’ sex, the form of the disease, or its duration.

In a Swedish study [34], patients were divided into four groups depending on their subjective perception of the severity of digestive symptoms. No evident correlation was found between symptom severity and HRQOL, irrespective of the type of questionnaire used (Short Form 36-SF-36 and Psychological General Well-Being index-PGWB). These observations are consistent with our findings. Unfortunately, other authors did not determine whether the subjective perception of symptoms affected patients’ QOL depending on age.

The concept of therapeutic education in chronic diseases has been known for years. Numerous educational organizations have been founded at the national and international level to prepare specialists in education. The World Health Organization (WHO) recommends education in such diseases as allergies, cancer, and endocrine disorders. Extending educational enterprises to other chronic disorders is also suggested [43].

The educational experience has increased awareness of the benefits related to implementing educational programs for functional disorders of the alimentary tract, including IBS. However, educational actions undertaken for irritable bowel syndrome have a limited scope and range. Single studies on the efficacy of education in IBS seem to confirm its positive impact.

The general effects of education for IBS patients have been proven to be beneficial. However, detailed analyses of the effects of the methods used are sometimes contradictory and none of them can be considered superior. Until now, the effect of individual education combined with nonpharmacological therapy in IBS patients has not been investigated. Thus, the current study is the first of that type.

A pilot study in Sweden [44] assessing the effect of education for IBS patients showed that the transmission of knowledge about the disease, dietary recommendations, lifestyle modifications, relaxation techniques, and methods of physical activity in organized support groups leads to a significant improvement in HRQOL, reduces symptom severity, and enriches patients’ knowledge of the disease. The study assessed single meetings of 12 patients with five specialists. The results were encouraging, and the study was continued with a larger group of patients.

The same researchers published the results of another study in 2010 [45], comparing two educational methods: (1) so-called self-education, in which patients received only written materials in the form of a brochure, and (2) education organized as “IBS school,” based on the theory of self-care and general nursing theories. The cognitive behavioral approach was applied [44].

A study conducted by Ringström et al. [44] revealed more substantial benefits of the IBS school as compared to the writing-type school. An improvement in QOL scores on the IBS-specific questionnaire (IBSQOL) was noted only in the case of the IBS school and referred to many domains assessed in the questionnaire. However, the authors found no significant difference between the two forms of education in the effect on QOL of IBS patients. A favorable impact was seen three and six months after education. These observations are largely consistent with our findings; also, in both studies, patients were recruited from a tertiary center. The results obtained in the current study and confirmed by other authors [44,45,46] show positive aspects of education for IBS patients, irrespective of the disease form.

The highly significant improvement in QOL among IBS patients found in the current study after individual educational–therapeutic sessions is the major achievement of this method of education. Tangible benefits in QOL were observed after individual therapy even though a generic questionnaire, which is less sensitive than a specific one, was used. Improvement was noted in all domains irrespective of the patients’ sex.

Interestingly, significant differences were found in six out of eight domains (PF, RP, SF, VT, RE, MH) between age groups after education compared to before. Differences in the PF and RP domains before education had been already noted, with the highest median for patients under the age of 35 and the lowest over the age of 50. The fact that the above difference persisted in two SF-36 domains in total QOL, in the physical component, and after education in the mental component indicates a smaller effect of the educational program for patients older than 50 compared to other age groups. Presumably, the above hypothesis is also confirmed by the QOL index, which in the current study was observed to decrease with age. Virtually all studies have focused on variables such as sex, disease form and duration, patient education, and marital status. However, the age of patients in a vast majority of studies is generally treated as the mean or median for the group, without clear differentiation between age categories and assessment of correlation of study parameters with age groups. The division into groups used in the current study allowed us to reveal a poorer effect of the therapy for patients over the age of 50. Whether this observation can be considered more universal and refer to a greater population of IBS patients should be the subject of a separate study.

In the current study, the severity of intestinal and parenteral symptoms was analyzed jointly. We did not assess single symptoms, such as severity and frequency of abdominal pain, flatulence, bowel movement disorders and changes in stool consistency, headaches, urination disorders, nausea, and heartburn. It can be assumed that in most cases, the study patients, recruited from a tertiary center, had a severe form of IBS, although the presence of mild and moderate forms cannot be excluded. This is due to the fact that in Poland such patients are relatively quickly referred to specialist care by family physicians.

Education was related to a significant decrease in the severity of symptoms experienced by patients. After the educational program, in only one case the severity of symptoms increased from mild to moderate. In the remaining cases, no change or improvement was noted, which evidently indicates a positive effect of education on this parameter. Taking into consideration the significantly elevated QOL scores of the study patients after the educational–therapeutic program, it cannot be ruled out that the increase may be indirectly (or directly) related to subjectively reduced symptoms due to the program.

Not all researchers have shown a beneficial effect of education on the QOL of IBS patients. Ringström et al. [45] observed that the use of written information in the form of a brochure (self-education) or additional educational sessions with groups of IBS patients did not significantly increase the QOL of those patients, which was assessed using both the generic SF-36 questionnaire and disease-specific IBSQOL.

Our program is similar to the one used in the IBS school in Goteborg [44] in its educational content. However, it differs in how the program was accomplished. In the IBS school, the program was carried out through workshops, and in our study we conducted direct sessions. Both forms have some advantages. Education in a group allows people to make new acquaintances, facilitates mutual understanding of disease-related problems, and improves motivation. Patients can share their own experiences, which can provoke discussions. On the other hand, a direct relationship between an IBS patient and an educator in an individual education program allows a patient-tailored approach, in which the subject matter and the level of difficulty can be adjusted to the patient’s needs and abilities.

Despite some similarities to the Swedish program, our program was to a large extent innovative, which was emphasized by study participants and confirmed by the literature. The main difference lay in the number of educators. In our program, education was conducted by one person (a nurse) in cooperation with a gastroenterologist. Another difference was the introduction of practical relaxation methods in the program.

The analysis of standardized questionnaires completed by patients after the educational program provides an objective evaluation of the program’s efficacy. An increase was observed in the subjective assessment of QOL as well as improvements in the physical and mental components, irrespective of the IBS form or its duration.

Patients who completed the educational program enriched their knowledge of the essence of the disease and learned about recommended methods of physical activity and stress-reducing techniques. The accomplished educational aims usually show a positive correlation with increased ability to cope with IBS symptoms in everyday life. Some patients admitted that they were encouraged by the program to talk about their disease-related problems with close relatives or other IBS patients, which gave them a different perspective. Undoubtedly, the educational actions increased trust in the medical staff engaged in the therapy.

Due to the permanently increasing costs of healthcare worldwide and in Poland, an economic analysis of expenses is taken into account when making decisions about treatment. Our educational therapeutic program did not require large financial expenditure.

This study has some limitations that must be acknowledged. The assessment of long-term effects of the individual program conducted in IBS patients should be repeated a year or two after its termination. The larger number of women in the study (79%) is a limitation. However, women suffer from IBS twice as often, but men are also a large proportion of patients with this disease. The use of a random sequence generator would have provided a much more reliable form of randomization. In future studies, the lack of blinding and a placebo control should be addressed.

## 5. Conclusions

In the group of patients with IBS, quality of life was found to be significantly lower than in the control group, despite long-term pharmacotherapy. An individual and varied educational–therapeutic program meeting patients’ expectations led to a significant improvement in the QOL of IBS patients and caused a subjective decrease in the severity of symptoms. Individual education together with nonpharmacological therapy can be considered an indispensable therapeutic component for patients with IBS, apart from pharmacotherapy.

## Figures and Tables

**Table 1:** Demographic characteristics of study groups.

Demographic Features	IBS Group with Constipation (*n* = 60)	IBS Group with Diarrhea (*n* = 20)	IBS Group Mixed (*n* = 70)	Total IBS Groups (*n* = 150)	Control Group (*n* = 100)	*p*
**Age**						
<35 Years	15	6	14	35	14	0.130
35–50 Years	13	5	29	47	37	
>50 Years	32	9	27	68	49	
Mean Age	49.45 ± 15.6	46.25 ± 18.07	47.5 ± 15.3	48.14 ± 15.77	50.79 ± 12.03	
Median	51.5	45	46	48.5	50	
**Gender**						
Men	9	6	17	32	20	0.422
Women	51	14	53	118	80	
**Marital Status**						
Single	13	7	14	34	13	0.305
Married	39	13	50	102	80	
Divorced	2	0	2	4	3	
Widowed	6	0	4	10	4	
**Education**						
Elementary	9	2	5	16	6	0.778
Vocational	7	2	7	16	10	
Secondary	28	10	33	71	55	
Higher	16	6	25	47	29	
**IBS Duration**						
0–5 Years	35	9	34	78	0	0.521
5–10 Years	18	9	23	50	0	
>10 Years	7	2	13	22	0	

IBS—irritable bowel syndrome. Based on a chi-square test of independent categorical variables.

**Table 2:** Effect of factors on subjective assessment of HQOL in physical and mental components of patients before and after education.

Factor		Physical Components of QOL		*p*	Mental Components of QOL		*p*
		Before Education M ± SD, Me (Min, Max)	After Education M ± SD, Me (Min, Max)		Before Education M ± SD, Me (Min, Max)	After Education M ± SD, Me (Min, Max)	
IBS Group		M 51.66 ± 19.79 95% CI 48.49–54.82	M 63.54 ± 19.51 95% CI 60.41–66.66	0.0001	M 45.49 ± 18.28 95% CI 42.56–48.41	M 59.24 ± 17.20 95% CI 56.48–61.99	0.0001
		Me 47.36 (min 36.66, max 69.72)	Me 68.15 (min 48.61, max 79.72)		Me 47.77 (min 31.25, max 60.00)	Me 61.06 (min 46.12, max 73.37)	
Control Group		M 85.17 ± 7.80 95% CI 83.64–86.69			M 79.22 ± 10.22 95% CI 77.21–81.22		
		Me 86.11 (min 81.25, max 90.59)			Me80.81 (min 73.06, max 86.56)		
*p*		0.0001			0.0001		
Age	<35 years	M 69.59 ± 17.65 95% CI 59.81–79.36	M 75.28 ± 12.19 95% CI 68.52–82.03	0.001	M 58.74 ± 18.45 95% CI 48.52–68.95	M67.47 ± 11.50 95% CI 61.10–73.83	0.0001
		Me 71.35 (min 62.46, max 82.67)	Me 78.12 (min 70.97, max 83.12)		Me 58.87 (min 49.06, max 1.54)	Me70.18 (min 59.77, max 74.75)	
	35–50	M 70.31 ± 20.29 95% CI 59.07–81.54	M 68.07 ± 18.79 95% CI 57.66–78.47	0.0001	M 63.20 ± 21.18 95% CI 51.46–74.93	M61.16 ± 18.15 95% CI 51.10–71.21	0.0001
		Me 75.00 (min 53.47, max 86.73)	Me 72.60 (min 52.01, max 83.61)		Me 67.37 min (50.12, max 82.0)	Me65.29 (min45.87, max73.37)	
	>50 years	M 59.30 ± 25.44 95% CI 50.48–68.11	M 54.69 ± 18.79 95% CI 48.17–61.20	0.0001	M 55.87 ± 24.83 95% CI 47.26–64.47	M 53.95 ± 17.09 95% CI 48.02–59.87	0.0001
		Me 57.98 (min 36.73, max 84.37)	Me 56.56 (min 37.81, max 69.2)		Me 61.00 (min 33.25, max 76.37)	Me 55.10 (min 38.68, max 67.93)	
*p*		0.0075	0.0001		0.0946	0.0007	
Sex	Male	M 67.86 ± 23.31 95% CI 59.78–75.93	M 64.94 ± 23.96 95% CI 56.66–73.24	0.0001	M 62.41 ± 20.00 95% CI 55.48–69.33	M 59.81 ± 20.98 95% CI 52.54–67.07	0.0001
		Me 73.61 (min 54.06, max 83.12)	Me 45.34 (min 50.97, max 88.75)		Me 65.14 (min 51.18, max 78.75)	Me 67.56 (min 48.25, max 73.87)	
	Women	M 64.29 ± 22.92 95% CI 60.15–68.42	M 63.16 ± 18.21 95% CI 53.87–66.44	0.0001	M 58.06 ± 23.32 95% CI 53.85–62.26	M 59.08 ± 16.12 95% CI 56.17–61.98	0.0001
		Me 71.73 (min 42.91, max 85.34)	Me 67.32 (min 47.56, max 78.75)		Me 60.58 (min 36.62, max 79.25)	Me 60.56 (min 45.87, max 72.66)	
*p*		0.2900	0.3020		0.3045	0.46000	
Place of Residence	Town	M 65.96 ± 23.31 95% CI 61.14–70.77	M 62.73 ± 19.86 95% CI 58.62–66.83	0.0001	M 59.73 ± 23.13 95% CI 54.95–64.50	M 58.55 ± 17.51 95% CI 54.93–62.16	0.0001
		Me 72.97 (min 43.4, max 86.11)	Me 67.63 (min 47.43, max 79.37)		Me 63.5 (min 38.31, max 80.25)	Me 60.06 (min 45.37, max 72.66)	
	Village	M 59.88 ± 20.83 95% CI 54.60–65.15	M 66.52 ± 18.12 95% CI 61.93–71.10	0.0001	M 54.85 ± 19.95 95% CI 49.80–59.89	M 61.77 ± 15.98 95% CI 57.72–65–81	0.0001
		Me 63.75 (min 43.68, max 76.87)	Me 69.33 (min 55.10, max 79.86)		Me 55.58 (min 38.25, max 67.62)	Me 67.25 (min 51.33, max 73.5)	
*p*		0.0701	0.4341		0.1408	0.3240	
Constipation group		M 45.85 ± 18.74 95% CI 41.10–50.59	M 59.94 ± 19.22 95% CI 55.07–64.80	0.0001	M 42.24 ± 16.87 95% CI 37.97–46.50	M 56.21 ± 14.63 95% CI 52.50–59.91	0.0001
		Me 39.37 (min 34.58, max 63.88)	Me 67.72 (min 43.36, max 76.79)		Me 37.79 (min 28.06, max 58.12)	Me 55.79 (min 45.62, max 68.25)	
Mixed group		M 55.99 ± 20.43 95% CI 51.20–60.77	M 65.61 ± 20.84 95% CI 60.72–70.49	0.0001	M 47.59 ± 18.73 95% CI 43.20–51.97	M 61.25 ± 19.13 95% CI 56.76–65.73	0.0001
		Me 56.90 (min 41.25, max 71.59)	Me 69.51 (min 52.01, max 83.75)		Me 50.29 (min 33.75, max 61.0)	Me 67.75 (min 49.70, max 74.37)	
Diarrhea group		M 53.51 ± 16.87 95% CI 45.61–61.40	M 67.11 ± 13.73 95% CI 60.68–73.53	0.0001	M 47.91 ± 20.15 95% CI 45.51–52.47	M 61.29 ± 16.64 95% CI 53.50–69.07	0.0001
		Me 52.77 (min 40.17, max 69.06)	Me 69.33 (min 55.13, max 77.04)		Me 50.25 (min 33.25, max 62.62)	Me 63.62 (min 49.02, max 73.56)	
*p*		0.0103	0.1121		0.2084	0.0788	
IBS Duration	0–5 years	M 51.77 ± 19.07 95% CI 47.53–56.00	M 63.40 ± 18.83 95% CI 59.22–67.57	0.0001	M 46.87 ± 17.55 95% CI 42.97–50.76	M 60.62 ± 16.25 95% CI 57.01–64.63	0.0001
		Me 48.33 (min 37.33, max 70.62)	Me 68.19 (min 47.56, max 78.75)		Me 50.06 (min 33.25, max 61.00)	Me 66.45 (min 49.70, max 73.00)	
	5–10 years	M 48.55 ± 20.03 95% CI 42.99–54.10	M 60.99 ± 21.29 95% CI 55.08–66.89	0.0001	M 42.10 ± 17.15 95% CI 37.34–46.85	M 55.23 ± 17.89 95% CI 50.27–60.18	0.0001
		Me 44.09 (min 35.90, max 64.72)	Me 66.59 (min 46.80, max 79.86)		Me 38.41 (min 25.87, max 55.66)	Me 56.35 (min 43.37, max 68.87)	
	>10 years	M 57.98 ± 21.09 95% CI 48.62–67.33	M 69.84 ± 16.87 95% CI 62.35–77.32	0.0036	M 48.33 ± 22.64 95% CI 38.29–58.36	M 63.25 ± 17.86 95% CI 55.32–71.17	0.0002
		Me 56.7 (min 37.98, max 76.87)	Me 72.39 (min 59.16, max 81.50)		Me 44.56 (min 31.25, max 63.5)	Me 63.77 (min 46.25, max 78.75)	
*p*		0.2613	0.2747		0.2443	0.0880	

ANOVA ranked Kruskal-Wallis test and a Wilcoxon paired test were used, *p—p* value.

**Table 3:** Changes in severity of symptoms assessed by visual analogue scale (VAS) by IBS patients.

Complaints before Educational Program	Complaints after Educational Program					Total
	No Symptoms	Low Severity	Moderate Severity	High Severity	Maximum Severity	
No Symptoms	6 (4)	0 (0)	0 (0)	0 (0)	0 (0)	6 (4)
Low Severity	7 (5)	32 (21)	1 (1)	0 (0)	0 (0)	40 (27)
Moderate Severity	8 (5)	48 (32)	10 (7)	0 (0)	0(0)	66 (44)
High Severity	2 (1)	18 (12)	12 (8)	0 (0)	0 (0)	32 (21)
Maximum Severity	1 (1)	1 (1)	3 (2)	1 (1)	0 (0)	6 (4)
Total	24 (16)	99 (66)	26 (17)	1 (1)	0 (0)	*p* = 0.0001

## Supplementary Materials

The following are available online at https://www.mdpi.com/1660-4601/17/12/4230/s1, Table S1: comparison of subjectively perceived quality of life in SF-36 domains for the control group compared to the IBS group. Table S2: comparison of subjectively perceived quality of life in SF-36 domains for IBS patients before and after education.
