# Supplementary material for: The Impact of an Individual Educational Program on the Quality of Life and Severity of Symptoms of Patients with Irritable Bowel Syndrome
Source: Int J Environ Res Public Health. 2020 Jun 13;17(12):4230. doi: 10.3390/ijerph17124230 (PMC7344839; doi:10.3390/ijerph17124230)
Supplement: Supplementary file 1 [file ijerph-17-04230-s001.pdf]

International Journal of
Environmental Research and Public Health

MDPI

Supplementary Table 1. Comparison of subjectively perceived quality of life in SF-36 domains for the control group compared to the IBS group.

Control Group		PF	RP	BP	GH	VT	SF	RE	MH	HP (QOL Index)
IBS group *p*		0.001	0.001	0.001	0.001	0.001	0.001	0.001	0.001	0.001
Men *p*		0.001	0.001	0.001	0.001	0.001	0.001	0.001	0.001	0.001
Women *p*		0.001	0.001	0.001	0.001	0.001	0.001	0.001	0.001	0.001
Age	< 35 years *p*	0.769	0.022	0.001	0.001	0.001	0.012	0.001	0.001	0.001
	35–50 years *p*	0.001	0.001	0.001	0.001	0.001	0.001	0.001	0.001	0.018
	> 50 years *p*	0.001	0.001	0.001	0.001	0.001	0.001	0.001	0.001	0.001

Mann-Whitney U-test was used for group comparisons (control group vs. IBS), *p*—*p* value. IBS—irritable bowel syndrome; PF—physical functioning; SF—social functioning; RP—role physical; RE—role emotional; BP—bodily pain; GH—general health; VT—vitality; MH—mental health.

Supplementary Table 2. Comparison of subjectively perceived quality of life in SF-36 domains for IBS patients before and after education.

Patients		PE	RP	BP	GH	VT	SF	RE	MH	HP (QOL Index)
IBS group *p*		0.001	0.001	0.001	0.001	0.001	0.001	0.001	0.001	0.001
Men *p*		0.014	0.007	0.001	0.001	0.007	0.123	0.001	0.001	0.001
Women *p*		0.001	0.001	0.001	0.001	0.001	0.001	0.001	0.001	0.001
Age	< 35 years *p*	0.042	0.003	0.001	0.001	0.001	0.014	0.001	0.001	0.001
	35–50 years *p*	0.001	0.001	0.001	0.001	0.001	0.175	0.001	0.001	0.001
	> 50 years *p*	0.001	0.001	0.001	0.001	0.001	0.001	0.001	0.001	0.001

Wilcoxon paired test was used for IBS group comparisons (before and after education), *p*—*p* value. IBS—irritable bowel syndrome; PF—physical functioning; SF—social functioning; RP—role physical; RE—role emotional; BP—bodily pain; GH—general health; VT—vitality; MH—mental health.
